# Effects of reduced gag cleavage efficiency on HIV-1 Gag-Pol package

**DOI:** 10.1186/s12866-022-02503-3

**Published:** 2022-04-09

**Authors:** Yi-Ru Lin, Shih-Ming Chu, Fu-Hsien Yu, Kuo-Jung Huang, Chin-Tien Wang

**Affiliations:** 1 Institute of Clinical Medicine, National Yang Ming Chiao Tung University School of Medicine, 112 Taipei, Taiwan; 2 Division of Clinical Research, Department of Medical Research, Taipei Veterans General Hospital, Taipei, Taiwan

**Keywords:** HIV-1, Protease, Gag, Gag-Pol, Gag cleavage, Virus assembly

## Abstract

**Background:**

HIV-1 pol, which encodes enzymes required for virus replication, is initially translated as a Gag-Pol fusion protein. Gag-Pol is incorporated into virions via interactions with Gag precursor Pr55^*gag*^. Protease (PR) embedded in Gag-Pol mediates the proteolytic processing of both Pr55gag and Gag-Pol during or soon after virus particle release from cells. Since efficient Gag-Pol viral incorporation depends on interaction with Pr55^*gag*^ via its N-terminal Gag domain, the prevention of premature Gag cleavage may alleviate Gag-Pol packaging deficiencies associated with cleavage enhancement from PR.

**Results:**

We engineered PR cleavage-blocking Gag mutations with the potential to significantly reduce Gag processing efficiency. Such mutations may mitigate the negative effects of enhanced PR activation on virus assembly and Gag-Pol packaging due to an RT dimerization enhancer or leucine zipper dimerization motif. When co-expressed with Pr55^*gag*^, we noted that enhanced PR activation resulted in reduced Gag-Pol cis or trans incorporation into Pr55^*gag*^ particles, regardless of whether or not Gag cleavage sites within Gag-Pol were blocked.

**Conclusions:**

Our data suggest that the amount of HIV-1 Gag-Pol or Pol viral incorporation is largely dependent on virus particle production, and that cleavage blocking in the Gag-Pol N-terminal Gag domain does not exert significant impacts on Pol packaging.

**Supplementary information:**

The online version contains supplementary material available at 10.1186/s12866-022-02503-3.

## Background

The HIV-1 structural protein Gag precursor Pr55^*gag*^ is capable of self-assembly into virus particles [1]. Essential viral enzymes for HIV-1 replication—including protease (PR), reverse transcriptase (RT), and integrase (IN)—are encoded by the *pol* gene [2]. Pol is initially translated as a Pr160^*gag − pol*^ fusion polyprotein. Both Pr55^*gag*^ and Gag-Pol are translated from the same mRNA template. A ribosomal shift event occurs at a frequency of approximately 5% during Gag translation, resulting in a Gag-Pol/Gag expression ratio of around 1:20 [3]. It is believed that activated PR embedded in Gag-Pol mediates Pr55^*gag*^ and Gag-Pol cleavage during virus release from cell surfaces. Pr55^*gag*^ cleavage yields four major products: matrix (MA; p17), capsid (CA; p24), nucleocapsid (NC; p7) and the C-terminal p6^*gag*^ domain [4]. Within Gag-Pol, p6^*gag*^ is truncated and replaced with a transframe region labeled p6^*pol*^ or p6*. Gag-Pol cleavage yields PR, RT and IN in addition to Gag products. The PR-mediated processing of Pr55^*gag*^ and Gag-Pol (referred to as virus maturation) is necessary for acquiring viral infectivity [5–8].

By itself, Gag-Pol is incapable of budding from cells as virus-like particles (VLPs), likely due to the absence of a p6^*gag*^ budding domain [9]. Data from several studies indicate that Gag-Pol is packaged into virus particles via interaction with Pr55^*gag*^ [10–13]. PR functions as a homodimer, and interactions between Gag-Pol molecules are thought to facilitate PR activation via the promotion of PR dimerization [1]. PR overexpression from altering the Gag-Pol/Gag expression ratio is known to markedly reduce virus yields due to enhanced Pr55^*gag*^ cleavage [14, 15]. Further, increased PR or Gag-Pol dimerization can trigger premature PR activation, resulting in significantly reduced virus yields due to premature Pr55^*gag*^ cleavage prior to virus particle assembly. This idea is supported by evidence indicating that treatment with efavirenz (EFV, an RT dimerization enhancer) or the insertion of a leucine zipper dimerization motif at the PR N- or C-terminus enhances Pr55^*gag*^ cleavage efficiency, leading to substantial reductions in virus yields [16–19]. Increases in retroviral PR or Gag-Pol expression frequently result in reduced virus yields due to enhanced Gag cleavage. However, one research team has reported that avian sarcoma leukosis virus PR is expressed in a Gag-PR context at a level equivalent to that of Gag [20]. This suggests the possible involvement of unidentified factors in protease activation regulation.

PR autocleaving triggered by PR dimerization results in the release of free PR for the subsequent cleaving of Gag-Pol and Gag precursors [21]. Results from an in vitro kinetics study of HIV-1 Gag-Pol processing by PR in trans suggest that initial cleaving at p2/NC is followed by cleaving at MA/CA [22]. Other Pr55^*gag*^ cleavage studies also suggest that initial cleaving occurs between the p2 space peptide and NC protein [5, 23–28]. Cleavage occurs at the MA/CA and p1/p6 sites following initial cleavage [5, 28]. It is unknown whether PR simultaneously cleaves both Gag-Pol (cis and trans) and Pr55^*gag*^ (trans), or if PR embedded in Gag-Pol cleaves Gag-Pol before cleaving Pr55^*gag*^. Given that both PR activation during virus assembly and Gag-Pol incorporation into virus particles are largely dependent on interactions between the Gag-Pol N-terminal Gag domain and Pr55^*gag*^, it is likely that Gag-Pol viral incorporation decreases following the premature cleaving of Gag from Gag-Pol. To test this idea, we created several mutations to block MA/CA and p2/NC cleavage sites within Gag-Pol and/or Pr55^*gag*^, and found that cleavage site blocking mitigated the negative impacts of enhanced PR activation on virus production. According to results from co-transfection experiments, there was no significant difference in the efficiency of Gag-Pol trans incorporation into Pr55^*gag*^ particles between wt Gag-Pol and Gag-Pol containing blocking mutations at MA/CA and p2/NC. Our data suggest that blocking Gag cleavage sites within Gag-Pol did not ameliorate the reduction in Gag-Pol packaging that resulted from enhanced Gag-Pol autocleavage, and that Gag-Pol packaging level is largely dependent on Gag virus particle production.

## Results

### Expression and assembly of HIV-1 gag cleavage-deficient mutants

**Fig. 1:**
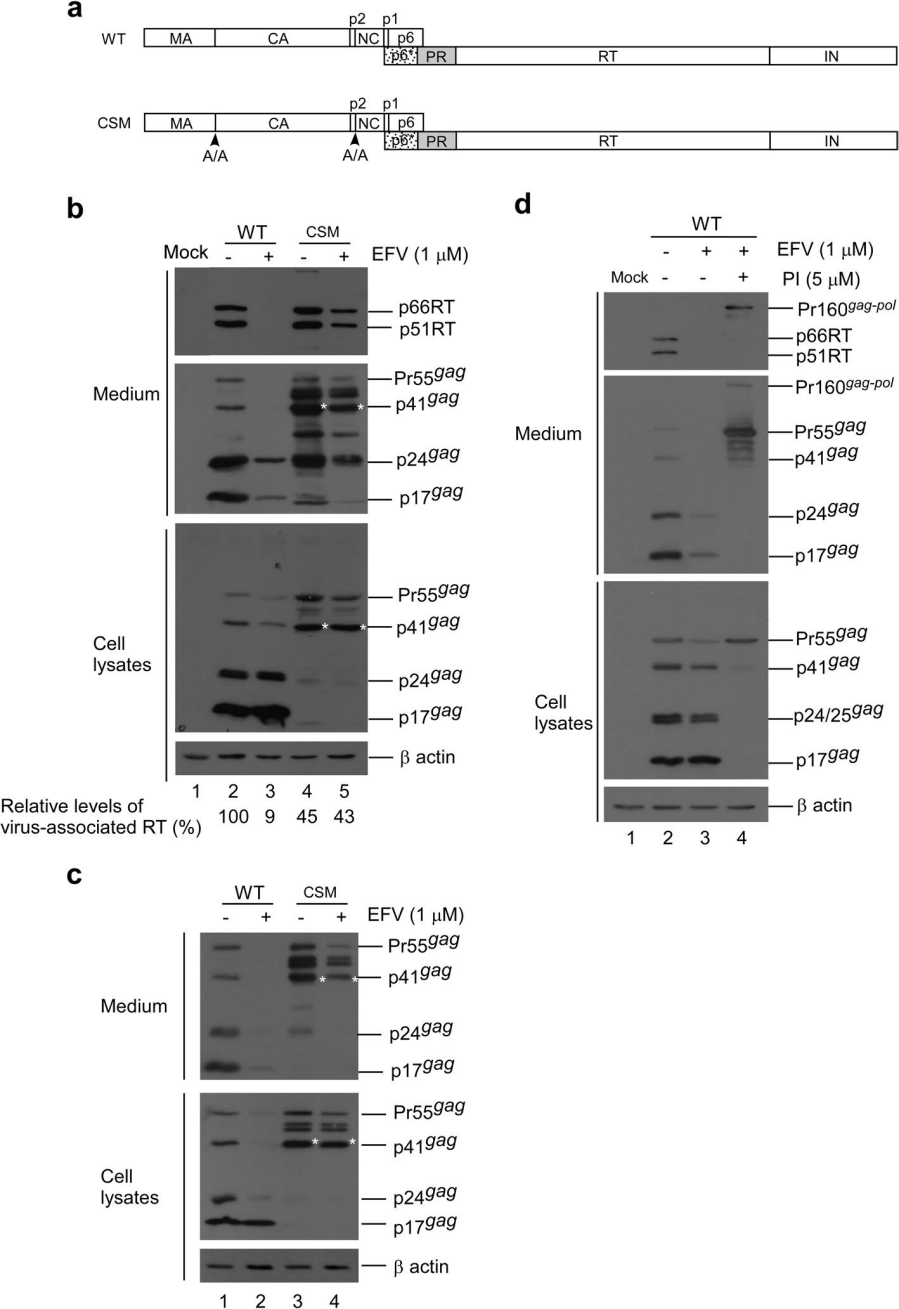
Effects of Gag cleavage site mutations on virus assembly and processing. **a** Schematic representations of HIV-1 Gag and Gag-Pol expression constructs. Indicated are the HIV-1 Gag protein domains MA (matrix), CA (capsid), p2, NC (nucleocapsid), p1 and p6, and pol-encoded p6*, PR, RT and IN. Arrowheads indicate where alanines (A/A) were substituted for amino acid residues at the MA/CA (Y/P) and p2/NC (M/M) junctions. **b**-**d** Enhanced PR activation by efavirenz (EFV) reduced virus yields and Gag-Pol packaging. 293T cells were transfected with designated constructs. At 18 h post-transfection, transfectants were equally split, placed on two plates, and either treated or mock-treated with 1 µM EFV. At 4 h post-treatment, culture supernatants were removed and replaced with medium containing EFV with or without an HIV-1 protease inhibitor (panel **d**). Shown is a representative immunoblot from two independent experiments. Cells and supernatants were harvested for Western immunoblot analysis 48 h after medium replacement. HIV-1 Gag proteins were probed with anti-p24CA and anti-p17MA monoclonal antibodies. Anti-RT serum was used to detect RT. Indicated are positions of the RT p66 and p51 subunits, Pr55^*gag*^, p4^1*gag*^, p24^*gag*^ and p17^*gag*^

To test the effects of Gag cleavage on virus assembly and Gag-Pol packaging, a mutant (designated CSM) with amino acid substitution mutations was engineered to block PR cleaving at p2/NC and MA/CA (Fig. 1a). Since Gag-Pol incorporation depends on interaction with Pr55^*gag*^, and since Gag cleavage efficiency can affect virus particle production, we used medium-associated Gag—specifically, Gag precursor Pr55^*gag*^, incompletely processed p41^*gag*^, and/or mature p24^*gag*^—as a virus production marker. Data for transient expression in 293T cells indicate significant reductions in the CSM cellular products p24^*gag*^ and p17^*gag*^ compared to those of a wt (Fig. 1b, lower panel, lane 4 vs. lane 2; supplementary Fig. 1b), confirming PR cleavage inhibition at the p2/NC and MA/CA junctions. The observation that CSM exhibited virus-associated p24^*gag*^ at a level comparable to that of the wt is likely due, at least in part, to western blot overexposure (Fig. 1b, middle panel, lane 4 vs. lane 2). In a repeat experiment we found that the level of CSM virus-associated p24^*gag*^ was noticeably lower than that of the wt (Fig. 1c, upper panel, lane 3 vs. lane 1; supplementary Fig. 1c). As a result of this blocking, substantial amounts of intermediate or incompletely processed Gag were detected in CSM supernatant and cellular samples. A band migrating slightly faster than wt p41gag (MA-CA-p2) likely corresponded to the MA-CA molecular weight (Fig. 1b and c, asterisks). Bands migrating to positions between Pr55gag and p41gag likely corresponded to the MA-CA-p2-NC(-p1) molecular weight (Fig. 1b, upper panel, lanes 4–5, and Fig. 1c, lanes 3–4). Some of the aberrant p24^*gag*^-associated products may have resulted from cleavages at cryptic sites when MA/CA- and p2/NC-accessible sites were blocked. These partial Gag cleavage products might assemble and release as VLPs, which would be consistent with prior findings that Gag lacking p6 and/or NC domains is still capable of assembly and release as VLPs in 293T cells [29, 30].

### Effects of enhanced gag cleavage on virus assembly and Pol packaging

We posited that Gag-Pol dimerization facilitated by EFV might enhance Gag-Pol autocleaving, with the possibility of reduced virus-associated RT or Gag-Pol levels resulting (at least in part) from premature Gag cleavage due to the enhanced autocleaving effect. Treatment with 1 µM or 5 µM EFV was found to significantly reduce virus yields as a result of enhanced Pr55^*gag*^ cleavage [16, 31]. Accordingly, CSM virus particles might contain higher levels of RT or Gag-Pol when PR activation is enhanced. We treated wt and CSM transfectants with 1 μm EFV to test this possibility. Consistent with previously reported results [32], EFV treatment triggered a significant decrease in wt virus yield due to enhanced Pr55^*gag*^ cleavage efficiency (Fig. 1b. lane 3, 1c, lane 2 and 1d, lane 3, supplementary Fig. 1b-d). The Pr55^*gag*^ cleavage enhancement effect from EFV is PR activity-dependent, as evidenced by the detection of substantial amounts of Pr55^*gag*^ particles following treatment with an HIV-1 PR inhibitor (Fig. 1d, lane 4). In contrast, CSM virus yields were only moderately reduced following EFV treatment (Fig. 1b, lane 4 vs. lane 5), suggesting that blocking PR to induce cleavages at MA/CA and p2/NC can counteract the negative EFV effect on virus production. Similar to the decrease in virus production, virus-associated RT levels also declined following EFV treatment.

### Blocking Pr55^*gag*^cleavage mitigates negative impacts of Gag-Pol overexpression on virus yields and RT packaging

**Fig. 2:**
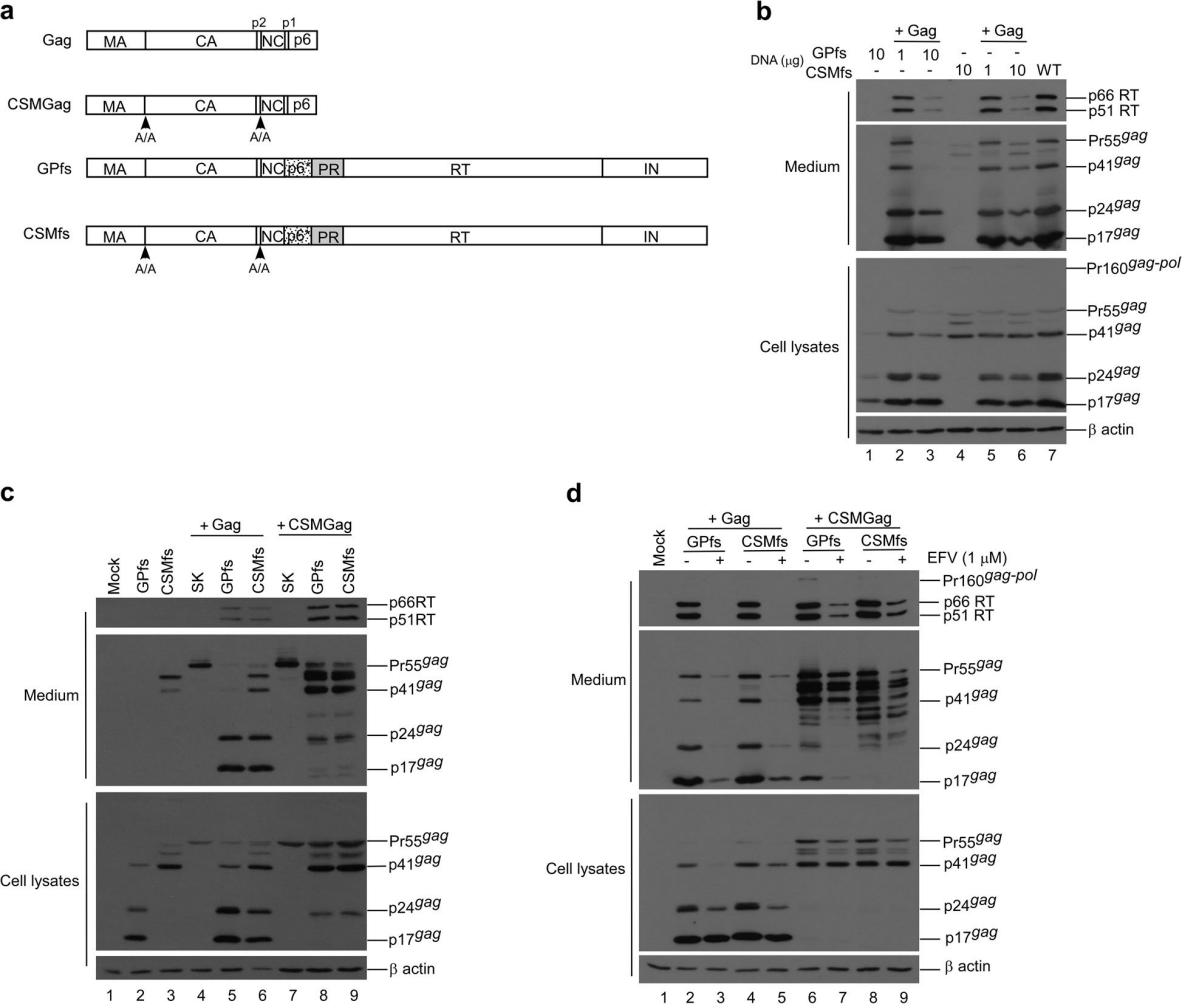
Effects of Gag cleavage mutation on Gag-Pol trans incorporation into virus particles. **a** Schematic representations of HIV-1 Gag and Gag-Pol expression constructs. Gag domains, pol-encoded products, and Gag cleavage site mutations are identical to those described in the Fig. 1 caption. GPfs and CSMfs both contained the same gag/pol frameshifting mutation to force Gag-Pol expression without Gag. **b** 293T cells were transfected with indicated amounts of GPfs or CSMfs plasmid DNA, either alone or with 10 µg Gag plasmid DNA at a ratio of 1:10 (lanes 2 and 5) or 1:1 (lanes 3 and 6). **c** 293T cells were transfected with 10 µg GPfs or CSMfs plasmid DNA, either alone or with 10 µg Gag (lanes 5–6) or CSMGag (lanes 8–9) plasmid DNA. Ten µg of pBluescript (SK) plasmid DNA was added to give a total of 20 µg DNA for each transfection. **d** 293T cells were co-transfected with either Gag (10 µg) or CSMGag (10 µg) plus either GPfs (1 µg) or CSMfs (1 µg) expression vectors. At 18 h post-transfection, transfectants were equally split, placed on two plates, and either treated or mock-treated with 1 µM efavirenz (EFV). After an additional 4 h (22 h post-transfection), culture supernatants were removed and replaced with medium containing EFV. Cells and supernatants were harvested for Western immunoblot analysis 48 h post-medium replacement. **b**-**d** Show is a representative immunoblot from three similar independent experiments

Since PR can access cleavage sites on Pr55^*gag*^ and Gag-Pol, and since reduced virus yields due to enhanced Pr55^*gag*^ cleavage are frequently associated with reduced Gag-Pol packaging, it is difficult to assess the impacts of Gag cleavage inhibition on Gag-Pol packaging when both Pr55^*gag*^ and Gag-Pol are expressed from the same plasmid, as observed in the CSM case. To address this challenge, we inserted the p2/NC and MA/CA cleavage site mutations into a Gag-Pol expression vector GPfs, and co-transfected the resulting construct (designated CSMfs) with either a wt- or CSM-containing Gag expression vector (Fig. 2a). The GPfs vector contains *pol* and *gag* in the same reading frame, resulting in the expression of Pr160^*gag − pol*^ only [15]. Our data indicate that virus-associated RT, p24^*gag*^, and p17^*gag*^ were readily detected when CSMfs or GPfs were co-transfected with a Pr55^*gag*^-expression vector (Gag) at a DNA ratio of 1:10 (Fig. 2b, lanes 2 and 5; supplementary Fig. 2b). This suggests that Gag cleavage blocking within Gag-Pol did not exert significant impacts on the ability of Gag-Pol to be incorporated into Pr55^*gag*^ particles in trans when the Gag-Pol/Pr55^*gag*^ expression ratio was close to matching physiological conditions. Unsurprisingly, noticeable reductions in virus-associated Gag and RT resulted when a Pr55^*gag*^-expressing vector was co-transfected with equal amounts of GPfs or CSMfs plasmid DNA (Fig. 2b lanes 3 and 6 and Fig. 2c lanes 5–6).

These data are in agreement with previously reported findings that Gag-Pol overexpression markedly reduces VLP yields. We noted that either GPfs or CSMfs overexpression with wt Gag (Pr55^*gag*^) resulted in barely detectable virus-associated RT accompanied by a reduction in virus-associated Gag. In contrast, virus-associated RT became readily detected with increasing virus-associated Gag when GPfs or CSMfs was co-expressed with CSMGag under the same condition (Fig. 2c, lanes 8–9 vs. lanes 5–6; supplementary Fig. 2c). No Gag products were detected when GPfs was expressed alone. However, p24^*gag*^-associated Gag products (likely corresponding to MA-CA-p2-NC and MA-CA) were detected in CSMfs transfectant supernatants (Fig. 2c middle panel, lane 2 vs. lane 3). This suggests that blocking Gag-Pol N-terminal Gag cleavage may yield incompletely processed Gag that is capable of forming VLPs, as observed in the CSM case (Fig. 1). Similar to the Gag-Pol overexpression scenario (Fig. 2c), virus yields decreased when PR activation was enhanced via EFV treatment (Fig. 2d; supplementary Fig. 2d). However, we did not observe significant differences between GPfs and CSMfs in terms of more efficient incorporation into wt Gag or CSMGag particles.

### Effects of PR activity inhibition on Pol packaging

**Fig. 3:**
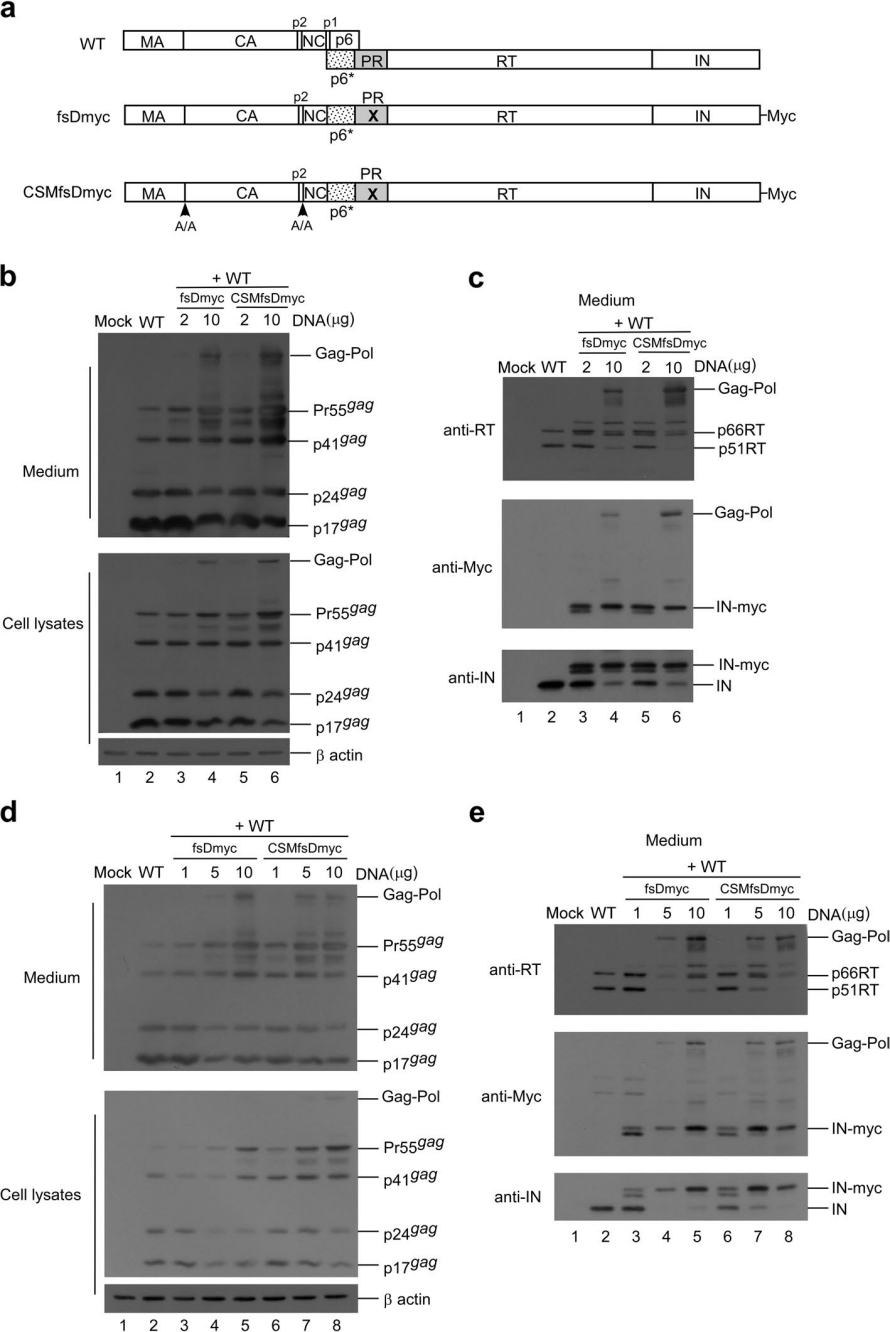
Incorporation of PR-defective Gag-Pol into wt virus particles. **a** Schematic representations of HIV-1 Gag and Gag-Pol expression vectors. Indicated are the HIV-1 Gag protein domains MA (matrix), CA (capsid), p2, NC (nucleocapsid), p1 and p6, and pol-encoded p6*, PR, RT and IN, all as described in the Fig. 1 caption. “X” denotes a substitution mutation at the PR catalytic Asp residue. The C-termini of constructs fsDmyc and CSMfsDmyc are attached with myc epitopes. **b**-**e** 293T cells were transfected with 10 µg of wt plasmid DNA plus the indicated amounts of fsDmyc or CSMfsDmyc plasmid DNA. At 48 h post-transfection, cells and supernatants were collected, prepared, and subjected to Western immunoblotting. Gag proteins were probed with anti-p24CA and anti-p17MA monoclonal antibodies. Panel c was derived from panel b following sequential probing with anti-RT, anti-Myc or anti-IN antibodies. Shown is a representative from three independent experiments

If Gag-Pol autocleaving occurs during or prior to viral incorporation, there is a possibility that PR-defective Gag-Pol may be packaged more efficiently than its PR-active counterpart. To test this possibility, PR-defective Gag-Pol with or without the CSM mutation was co-expressed with a wt. To distinguish PR-defective Gag-Pol from wt Gag-Pol, a Myc epitope was tagged at the C-terminus of PR-defective Gag-Pol and designated fsDmyc or CSMfsDmyc (Fig. 3a). Transient co-expression results indicate that increases in co-transfected amounts of these PR-defective Gag-Pol expression vectors led to increases in Gag and Gag-Pol precursors associated with moderate decreases of RT66/51 and integrase (IN) in supernatant samples (Fig. 3b-c, lanes 3–6, and Fig. 3d-e, lanes 3–8; supplementary Fig. 3b-e). These findings suggest that due to overexpression, PR-defective Gag-Pol is packaged at a higher frequency than wt Gag-Pol, and that the defective PR domain interferes with wt PR activity, resulting in the inefficient processing of Pr55^*gag*^ and Gag-Pol. Virus-associated IN and IN-myc levels were roughly equal when co-transfected PR-defective Gag-Pol plasmids were reduced to near-wt Gag-Pol expression levels (Fig. 3c, lanes 3 and 5). Combined, these data suggest that wt Gag-Pol is capable of mediating the processing of incorporated PR-defective Gag-Pol in trans, and that PR-defective Gag-Pol (with or without CSM) does not exhibit greater packaging efficiency compared to wt Gag-Pol when the two are co-expressed at approximately equal levels.

### Effects of enhanced Gag-Pol autocleavage on Pol packaging

In a previous report we described a Dp6*PR construct containing a duplicate p6*-PR with an inactivated proximal PR copy exhibiting a wt virus processing profile [33]. However, in that study virus-associated Gag was barely detectable when a leucine zipper (LZ) dimerization motif was substituted for the p6* and placed adjacent to the active PR. The severe assembly defect of this mutant (designated DWzPR) resulted from enhanced PR activation due to an LZ substitution for the deleted p6*, but a DWz/PR construct containing four C-terminal p6* residues in the deleted p6* region was found to counteract the LZ enhancement of PR activation [19]. We hypothesized that enhanced Gag-Pol autocleavage triggered by LZ may lead to reduced Pol viral incorporation, and that preventing Gag cleavage from Gag-Pol might retain that incorporation at the same level.

**Fig. 4:**
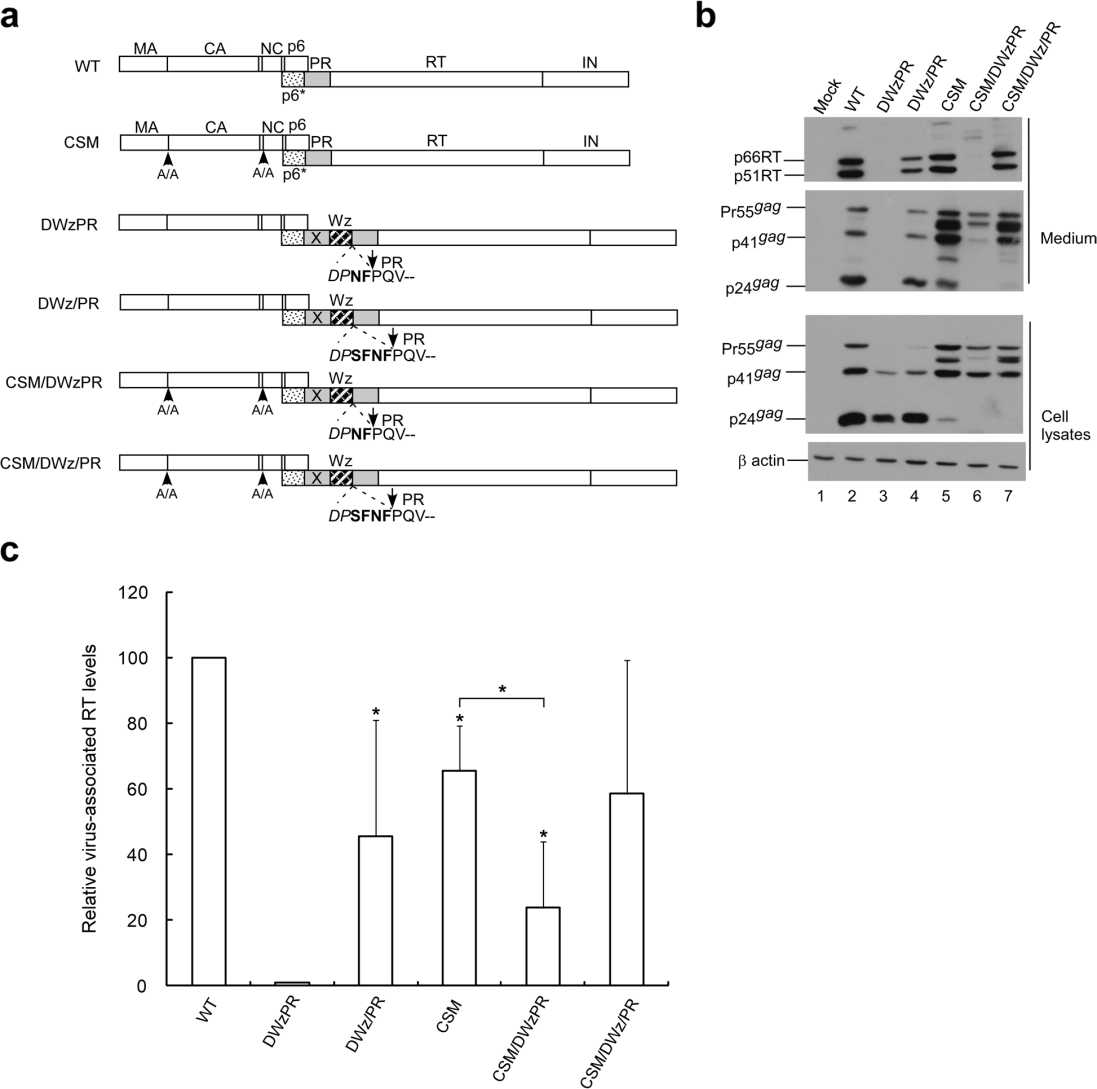
Virus yields and Gag-Pol packaging reduced by enhanced Gag-Pol autocleavage. **a** Schematic representations of HIV-1 Gag and Gag-Pol expression constructs. “X” denotes a PR-inactivated mutation. p6* adjacent to the active PR domain was replaced with a leucine zipper (LZ) as denoted by the striped (Wz) box. Remaining C-terminal p6* residues are in boldface. Arrows denote PR cleavage sites. Altered or additional residues are in italics. **b** 293T cells were transfected with designated constructs. Two days post-transfection, cells and supernatants were collected, prepared, and subjected to Western immunoblotting. **c** Relative levels of virus-associated RT. Levels of HIV-1 p24gag-associated Gag proteins Pr55, p41, and p24 and RT-associated Pol proteins Gag-Pol, Pol and p66/51RT were quantified by scanning immunoblot band densities. Ratios of total virus-associated RT versus Gag protein levels were calculated for each sample and normalized to the wt in parallel experiments. All data were obtained from three independent experiments. Error bars indicate standard deviation *p < 0.05

To test this possibility, a CSM mutation was cloned into DWzPR and DWz/PR, respectively yielding CSM/DWzPR and CSM/DWz/PR (Fig. 4a). Transient expression results indicate that DWzPR produced barely detectable virus-associated Gag, while DWz/PR virus-associated Gag products were readily detectable (Fig. 4b; supplementary Fig. 4b); this is consistent with previous reports [19, 34]. We found that compared to the wt transfectant, cellular Pr55^*gag*^ was barely detectable in DWzPR and DWz/PR transfectants (Fig. 4b lower panel, lanes 2–4), indicating enhanced PR-mediated Gag cleavage efficiency. Both DWzPR and DWz/PR exhibited barely detectable or noticeably reduced virus-associated RT compared to their CSM-containing counterparts (Fig. 4b upper panel, lanes 3–4 vs. lanes 6–7, and Fig. 4c).

**Fig. 5:**
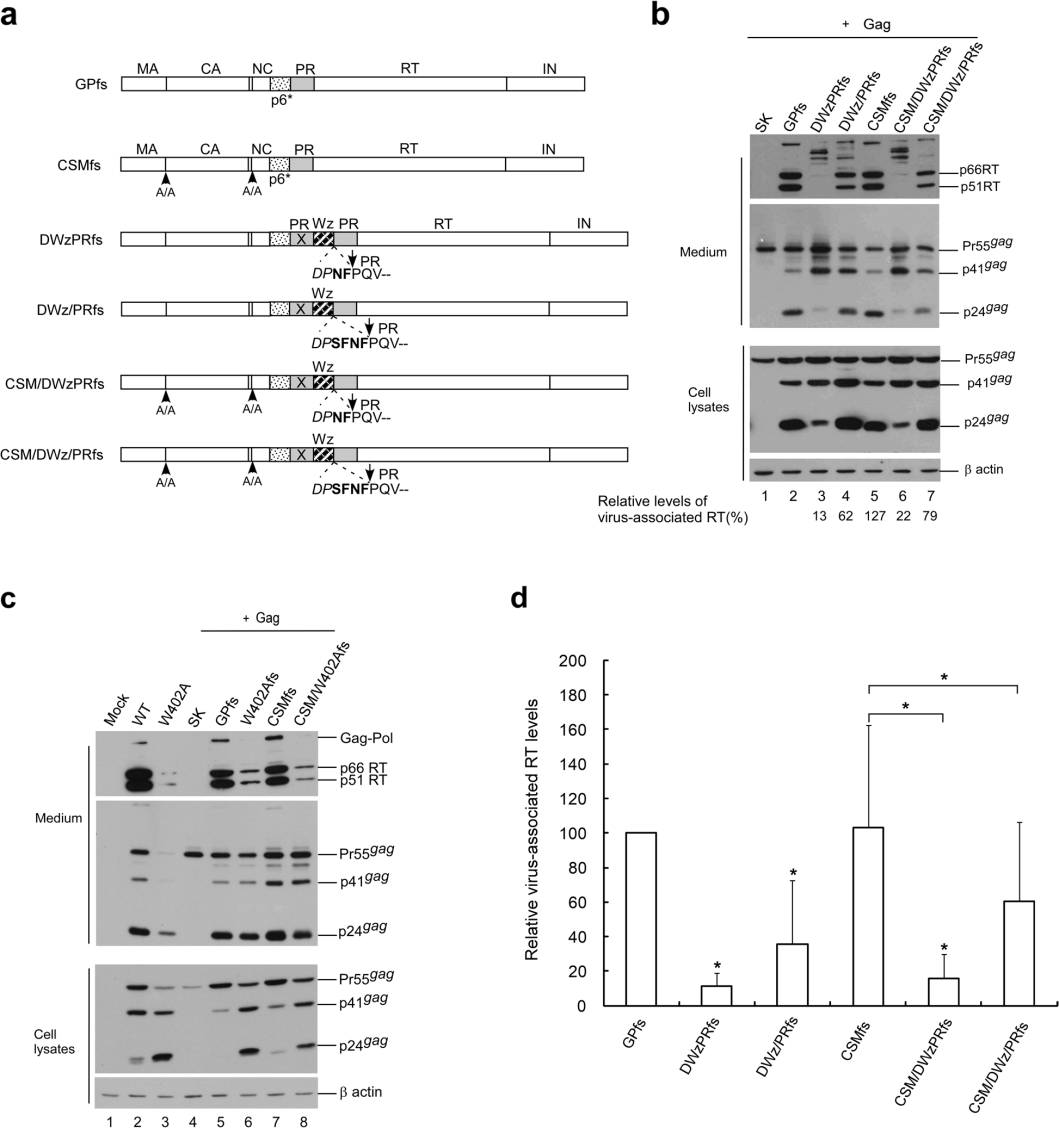
Enhanced Gag-Pol autocleavage reduced Gag-Pol trans incorporation into Gag particles. **a** Schematic representations of HIV-1 Gag and Gag-Pol expression constructs. Designated constructs are identical to those shown in Fig. 4a, but expressed in a gag/pol frameshift mutation backbone (GPfs). **b**-**c** 293T cells were transfect with WT or W402A or co-transfected with the designated construct plus a Pr55gag (Gag) expression vector at a plasmid DNA ratio of 1:10. SK denotes the plasmid pBluescript. At 48–72 h post-transfection, cells and supernatants were collected, prepared, and subjected to Western immunoblotting. Relative levels of virus-associated RT are indicated at bottom. **d** Relative levels of virus-associated RT were determined as described in the Fig. 4 caption. All data are from three independent experiments. Error bars indicate standard deviation *p < 0.05

The results shown in Fig. 4 suggest that blocking Gag cleavage can partly counteract the reduced Pol packaging incurred by the LZ replacement of p6*. However, Pol or Gag-Pol packaging is primarily determined by their respective ability to associate with Pr55^*gag*^. In addition to reducing VLPs, enhanced PR activation may reduce Gag-Pol viral packaging as a result of enhanced Gag-Pol autocleavage. Since it is difficult to assess the impact of enhanced Gag-Pol autocleavage incurred by LZ on Gag-Pol packaging when Gag cleavage sites within Pr55^*gag*^ and Gag-Pol are blocked, we assessed the degree of DWZPR and DWz/PR Gag-Pol incorporation into wt Pr55^*gag*^ particles. After cloning DWzPR and DWz/PR with or without the CSM mutation into the GPfs backbone (Fig. 5a), each resultant construct was co-transfected with the Pr55^*gag*^ expression vector. Our results indicate that the leucine zipper replacement of p6* significantly reduced RT-associated products in virus particles (Fig. 5b upper panel, lanes 3–4 and 6–7 and Fig. 5d; supplementary Fig. 5b). However, virus-associated RT levels in DWzPRfs and DWz/PRfs did not increase significantly when Gag cleavage sites were blocked (Fig. 5b lane 3 vs. lane 6 and lane 4 vs. lane 7; Fig. 5d). As an additional test, Pr55^*gag*^ was co-expressed with a W420A-containing GPfs construct with or without the CSM mutation. W402A (with an alanine substitution of the RT codon W402) was found to be severely assembly-defective due to enhanced PR activation triggered by the W402A mutation [35]. Consistent with Chiang et al.’s results, W402A exhibited markedly reduced VLP yields compared to the wt (Fig. 5c, lanes 2–3; supplementary Fig. 5c) and reduced Pol incorporation into Pr55^*gag*^ particles, regardless of whether or not the Gag-Pol contained CSM (Fig. 5c upper panel, lanes 5–8).

## Discussion

We found that amino acid substitutions at the MA/CA and p2/NC junctions significantly reduced Gag cleavage efficiency. We also noted readily detected virus-associated p66/51RT expressed by CSM, suggesting that Gag cleavage site blocking did not exert major impacts on PR cleaving at Pol substrate sites (Fig. 1). Gag-Pol viral incorporation depends on interaction with Pr55gag. Although partial Pr55^*gag*^ cleavage products are VLP assembly-competent, they are likely defective in their interactions with Gag-Pol, resulting in RT-deficient VLP assembly. This may have contributed, at least in part, to the significant reduction observed in CSM virus-associated RT compared to the wt (Figs. 1 and 4).

The results shown in Fig. 2 C suggest that blocking Gag cleavage could alleviate the negative impact of Gag-Pol overexpression on virus particle production and Pol packaging, whether or not the Gag-Pol contained the CSM mutation. Likewise, we observed that enhanced Gag-Pol autocleaving by EFV resulted in lower VLP yields associated with reduced Pol (RT) incorporation (Fig. 2d), regardless of the presence or absence of the CSM mutation. Similarly, enhanced Gag-Pol autocleavage triggered by the LZ replacement of p6* led to significantly reduced Pol packaging efficiency, regardless of whether or not the Gag-Pol domain contained cleavage site mutations at MA/CA and p2/NC (Fig. 5). These findings suggest that the degree of Gag-Pol incorporation into VLPs is largely determined by the level of Gag particle production, regardless of the presence or absence of CSM. Our results indicate enhanced Gag-Pol autocleaving following Gag-Pol over-expression, EFV treatment, the replacement of p6* with LZ, or an alanine substitution for W402, resulting in reduced virus-associated Gag and RT levels. The observation that enhanced PR activation led to significant and simultaneous reductions in VLP yields and Pol packaging suggests that PR, once activated, can immediately mediate Gag-Pol and Gag processing during virus assembly. Enhanced or premature Gag-Pol autocleavage may impede Gag-Pol viral incorporation, which in turn might partly account for the observed significant reduction in DWz/PR virus-associated RT (Fig. 4).

## Conclusions

Our results suggest that Gag cleavage blocking can mitigate the negative effect of enhanced PR-mediated Gag cleavage efficiency, thereby moderating Pol packaging linked with reduced virus yields. Enhanced Gag-Pol autocleavage or degradation due to premature or enhanced PR activation may explain, at least in part, the observed reduction in Pol packaging efficiency. However, cleavage blocking at MA/CA or p2/NC within Gag-Pol did not significantly mitigate the Pol packaging defect incurred by enhanced Gag-Pol autocleavage.

## Methods

### Plasmid construction

The parental HIV-1 proviral sequence in this study is HXB2 [36]. CSM was constructed via the recombination of two mutants, MA/CA and p2/NC, both of which contain alanine substitutions for PR substrate residues at the MA/CA (Tyr/Pro) and p2/NC (Met/Met) junctions. Alanine substitutions for Tyr/Pro at the MA/CA cleavage site were executed by megaprimer PCR [37] using a mutation-containing forward primer 5’-CAGGTCAGCCAAAATGCCGCTATTGTACAGAACAT-3’, a downstream reverse primer 5’-TTGGACCAACAAGGTTTCTGTC-3’ (nt. 1761-39), and an upstream forward primer 5’-GACTAGCGGAGGCTAGAAG-3’ (nt. 763 − 81). HIVgpt [38] served as a template. PCR-amplified fragments were digested with ClaI and SpeI and cloned into HIVgpt. Similarly, a p2/NC cleavage site mutation was created using a mutation-containing forward primer 5’-AACAAATTCAGCTACGATCGCTGCTCAGAGAGGCA-3’, a downstream reverse primer 5’-GGTACAGTCTCAATAGGGCTAATG-3’ (nt. 2577-51), and an upstream forward primer 5’-GGAACTACTAGTACCCTTCAGGAACAA-3’ (nt. 1500-27). Amplified fragments were digested with SpeI and BclI prior to ligation into HIVgpt. As previously described, GPfs contains Gag and Pol in the same reading frame due to a frameshift signal deletion [15]. Construct fsD is a PR-inactivated version of GPfs [15]. fsDmyc was created by the in-frame fusing of the myc-his tag coding sequence (XhoI-PmeI fragment from pcDNA3.1/myc-His A) to fsDSal containing a SalI site at the IN C-terminus. The recombination of fsDmyc and CSM generated CSMfsDmyc. DWzPR and DWz/PR were as previously described [19]. CSM recombined with DWzPR and DWz/PR yielded CSM/DWzPR and CSM/DWz/PR, respectively. All constructs located in the HIVgpt backbone [38] were confirmed by enzyme digestion or sequencing.

### Cell culture and transfection

293T cell line was purchased from Elabscience (catalogue no. EP-CL-0005). Cells were maintained in DMEM supplemented with 10% fetal calf serum. Confluent 293T cells were trypsinized, split 1:10, and seeded onto 10 cm plates 18–24 h prior to transfection. For each construct, 293T cells were transfected with 20 µg of plasmid DNA by calcium phosphate precipitation, with the addition of 50 µM chloroquine to enhance transfection efficiency. Culture medium and cells were harvested for protein analysis at 48–72 h post-transfection. When co-transfecting Pr55gag-expressing plasmids with Gag-Pol expression constructs at DNA ratios of 1:1 or 10:1, pBlueScript (SK) plasmid DNA was added to 10 or 15 µg of pGag to a final quantity of 20 µg.

### Western immunoblot analysis

Culture medium from transfected 293T cells were filtered (0.45 μm pore size) and centrifuged through 2 ml 20% sucrose in TSE (10 mM Tris-HCl, pH 7.5, 100 mM NaCl, 1 mM EDTA) containing 0.1 mM phenylmethylsulfonyl fluoride (PMSF) at 4 °C for 40 min at 274,000 × *g*. Next, viral pellets and cell lysates mixed with sample buffer were subjected to SDS 10% PAGE with 4–12% Bis-Tris gradient gels (NuPage Bis-Tris Mini Gels; Thermo Fisher Scientific), followed by immunoblotting as previously described [32]. HIV-1 Gag proteins were probed with anti-p24gag (mouse hybridoma clone 183-H12-5 C) and/or anti-p17gag (No. HB-8975; American Type Culture Collection, Rockville, MD) monoclonal antibodies from ascites. Rabbit antiserum or mouse anti-RT monoclonal antibodies served as primary antibodies for HIV-1 RT detection [39, 40]. Cellular β-actin was detected using a mouse anti-β-actin monoclonal antibody (Sigma) with either a sheep anti-mouse or a donkey anti-rabbit horseradish peroxidase (HRP)-conjugated secondary antibody (Jackson ImmunoResearch). Membrane-bound protein detection was performed using an enhanced chemiluminescent substrate (SuperSignal West Pico; Thermo Fisher Scientific).

### Statistical analysis

Differences between control (wt) and experimental (mutant) groups were assessed using Students t-tests. Data are expressed as mean ± standard deviation. Significance was defined as *p < 0.05, **p < 0.01, ***p < 0.001.

## Supplementary Information

**Additional file 1.** Original uncropped blot images for Figs. 1b-d.

**Additional file 2.** Original uncropped blot images for Figs. 2c-e.

**Additional file 3.** Original uncropped blot images for Figs. 3b-e.

**Additional file 4.** Original uncropped blot images for Fig. 4.

**Additional file 5.** Original uncropped blot images for Figs. 5b-c.

## Data Availability

The datasets used and/or analyzed during the current study available from the corresponding author on reasonable request.
