# Supplementary figures and images for: Effects of reduced gag cleavage efficiency on HIV-1 Gag-Pol package
Source: BMC Microbiol. 2022 Apr 9;22:94. doi: 10.1186/s12866-022-02503-3 (PMC8994222; doi:10.1186/s12866-022-02503-3)

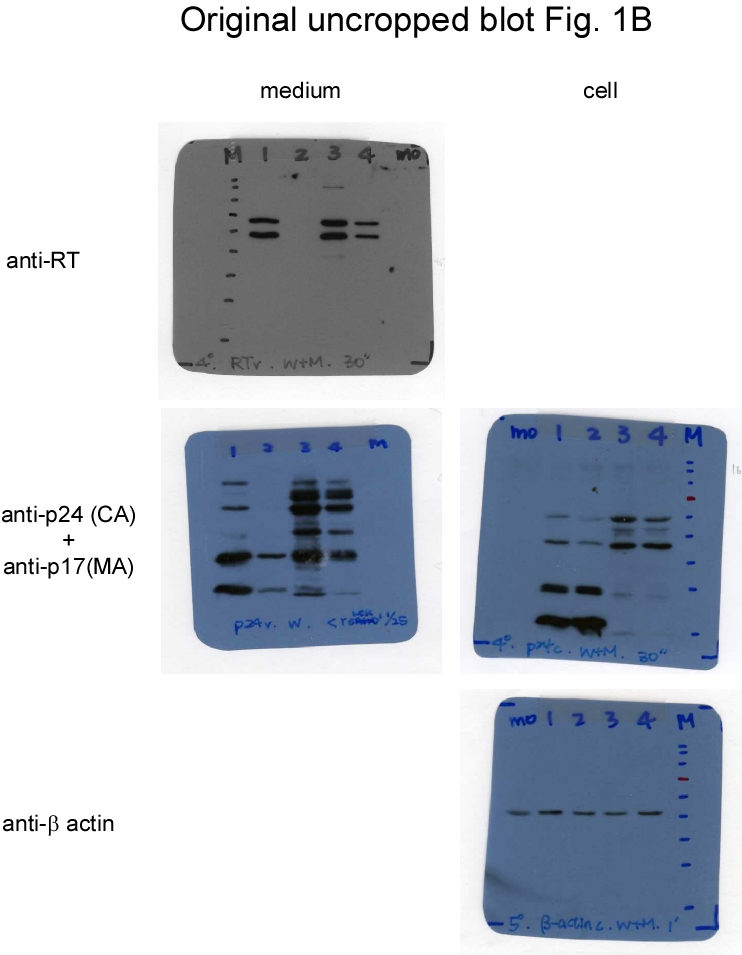

Supplement: Supplementary file 1 — Additional file 1. Original uncropped blot images for Figs. 1b-d. [file 12866_2022_2503_MOESM1_ESM.zip › Original uncropped blot Fig 1B.jpg]

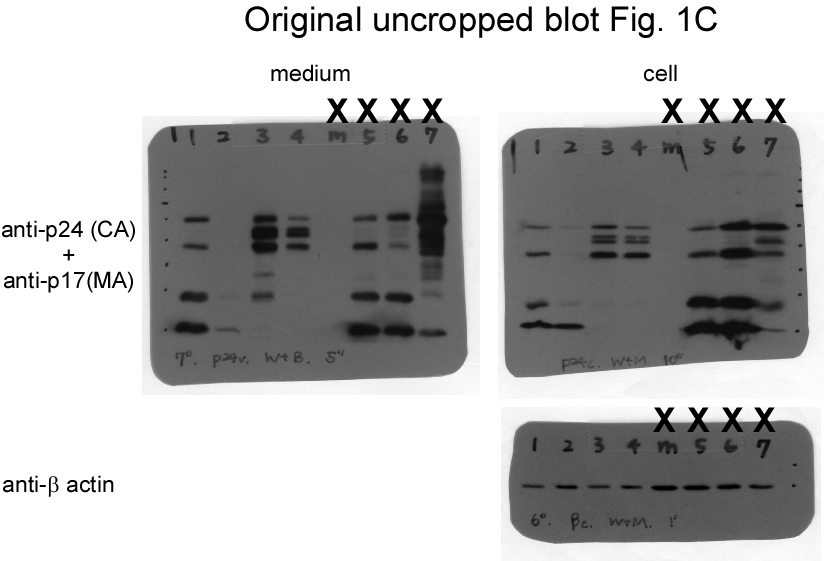

Supplement: Supplementary file 1 — Additional file 1. Original uncropped blot images for Figs. 1b-d. [file 12866_2022_2503_MOESM1_ESM.zip › Original uncropped blot Fig 1C.jpg]

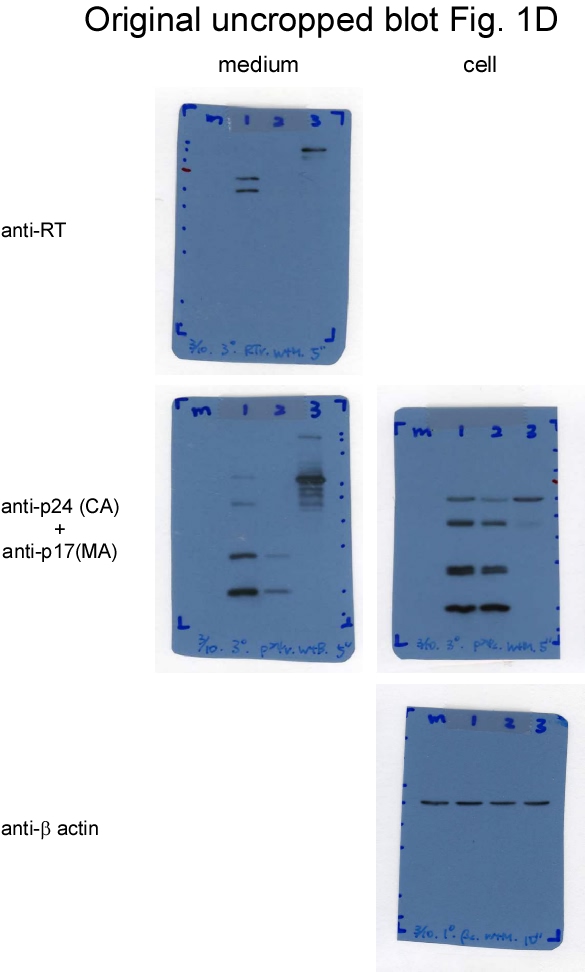

Supplement: Supplementary file 1 — Additional file 1. Original uncropped blot images for Figs. 1b-d. [file 12866_2022_2503_MOESM1_ESM.zip › Original uncropped blot Fig 1D.jpg]

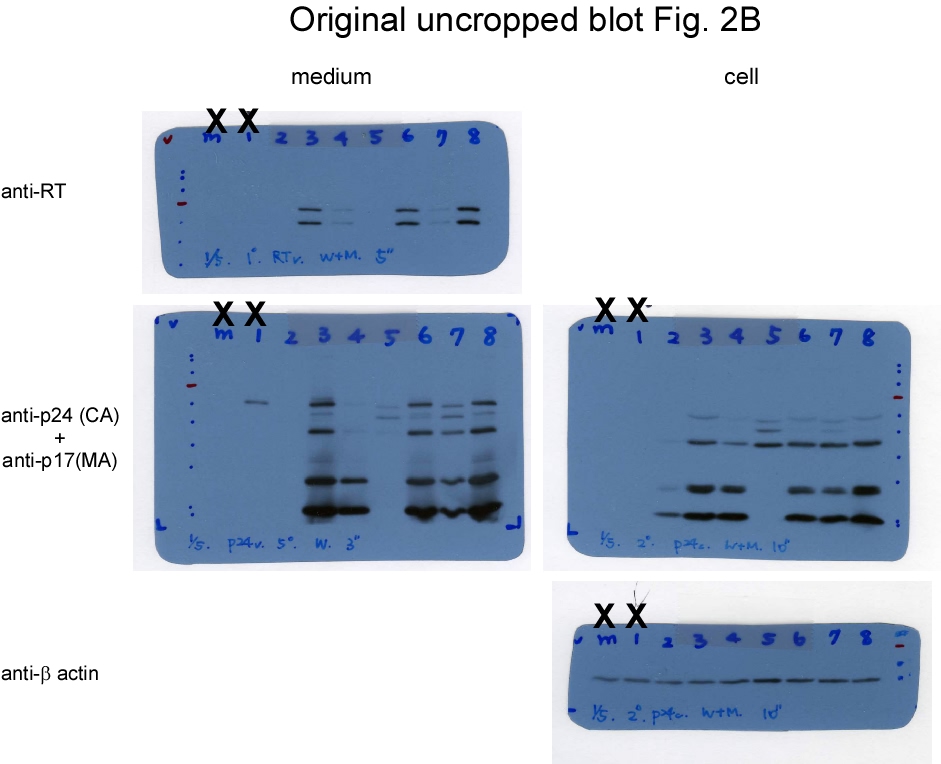

Supplement: Supplementary file 2 — Additional file 2. Original uncropped blot images for Figs. 2c-e. [file 12866_2022_2503_MOESM2_ESM.zip › Original uncropped blot Fig 2B.jpg]

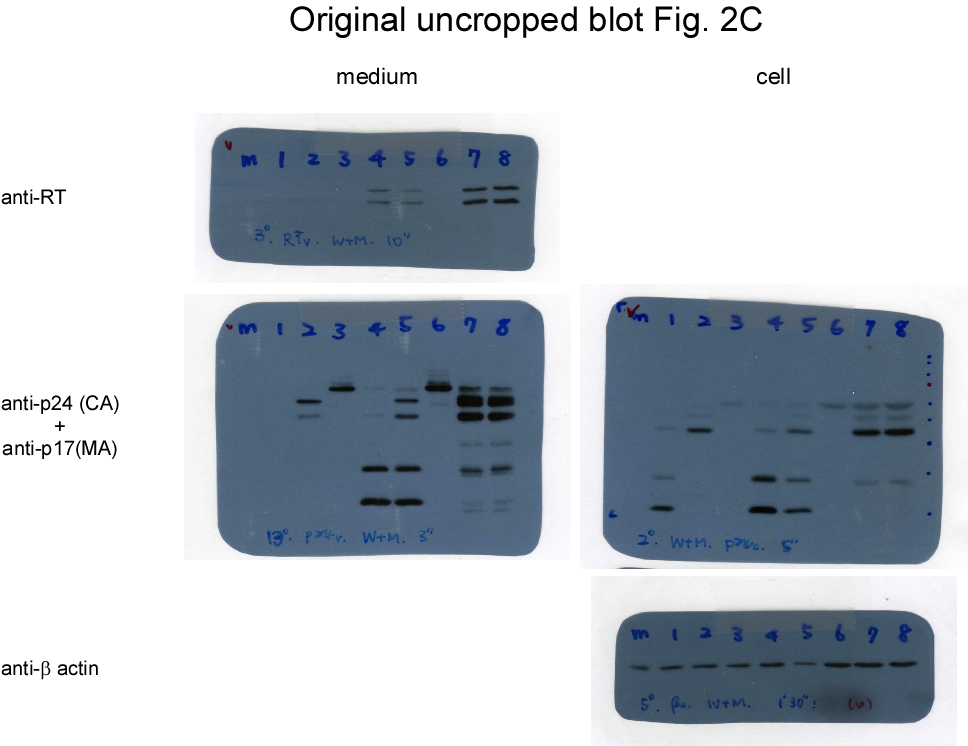

Supplement: Supplementary file 2 — Additional file 2. Original uncropped blot images for Figs. 2c-e. [file 12866_2022_2503_MOESM2_ESM.zip › Original uncropped blot Fig 2C.jpg]

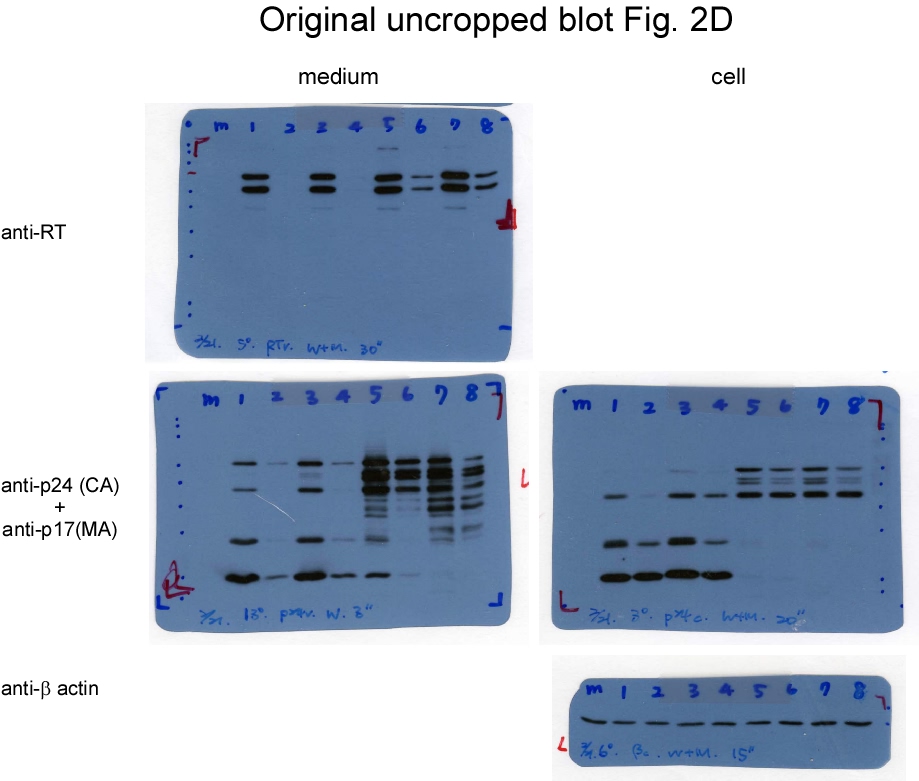

Supplement: Supplementary file 2 — Additional file 2. Original uncropped blot images for Figs. 2c-e. [file 12866_2022_2503_MOESM2_ESM.zip › Original uncropped blot Fig 2D.jpg]

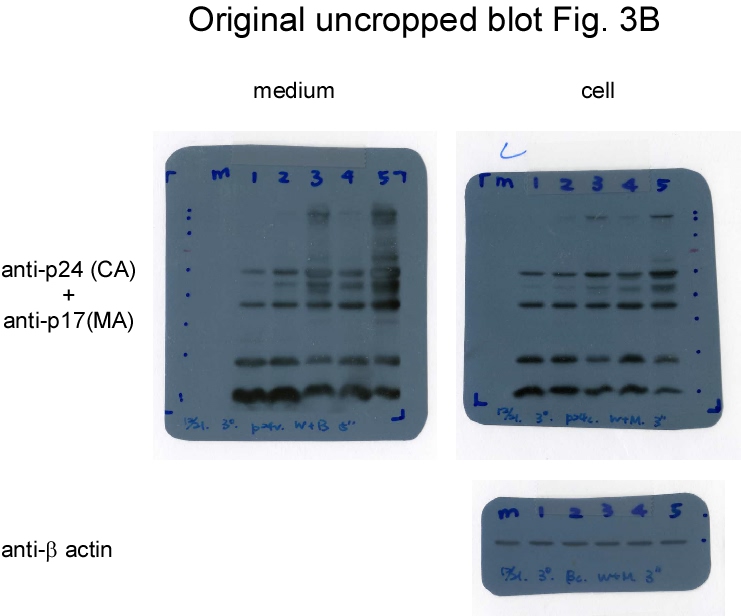

Supplement: Supplementary file 3 — Additional file 3. Original uncropped blot images for Figs. 3b-e. [file 12866_2022_2503_MOESM3_ESM.zip › Original uncropped blot Fig 3B.jpg]

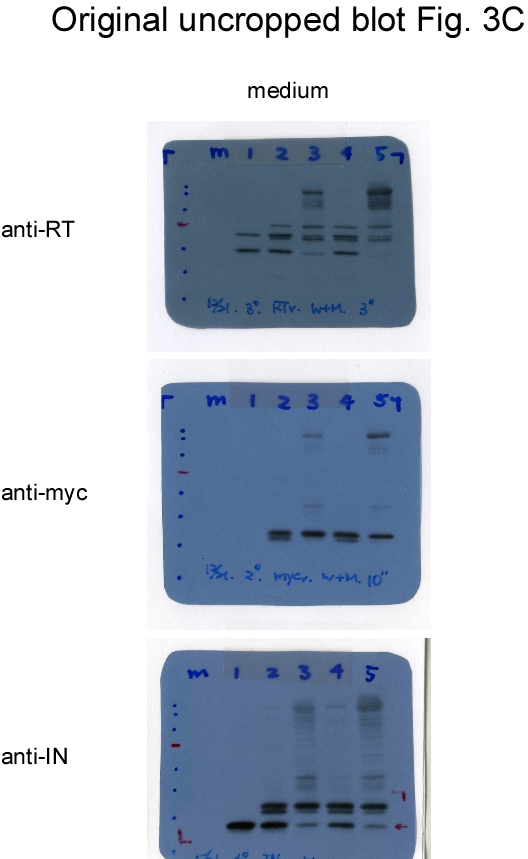

Supplement: Supplementary file 3 — Additional file 3. Original uncropped blot images for Figs. 3b-e. [file 12866_2022_2503_MOESM3_ESM.zip › Original uncropped blot Fig 3C.jpg]

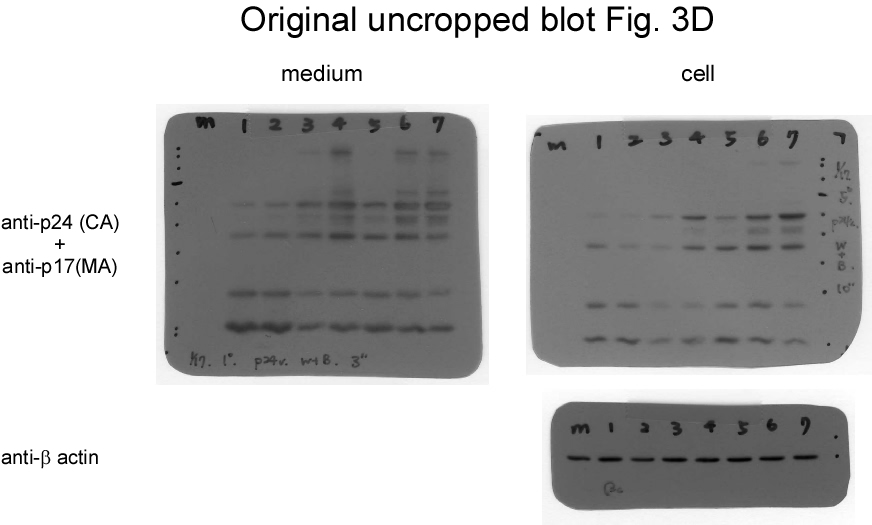

Supplement: Supplementary file 3 — Additional file 3. Original uncropped blot images for Figs. 3b-e. [file 12866_2022_2503_MOESM3_ESM.zip › Original uncropped blot Fig 3D.jpg]

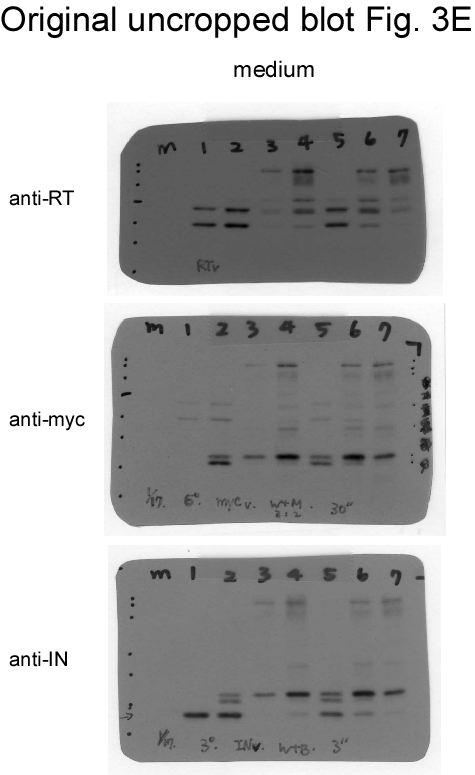

Supplement: Supplementary file 3 — Additional file 3. Original uncropped blot images for Figs. 3b-e. [file 12866_2022_2503_MOESM3_ESM.zip › Original uncropped blot Fig 3E.jpg]

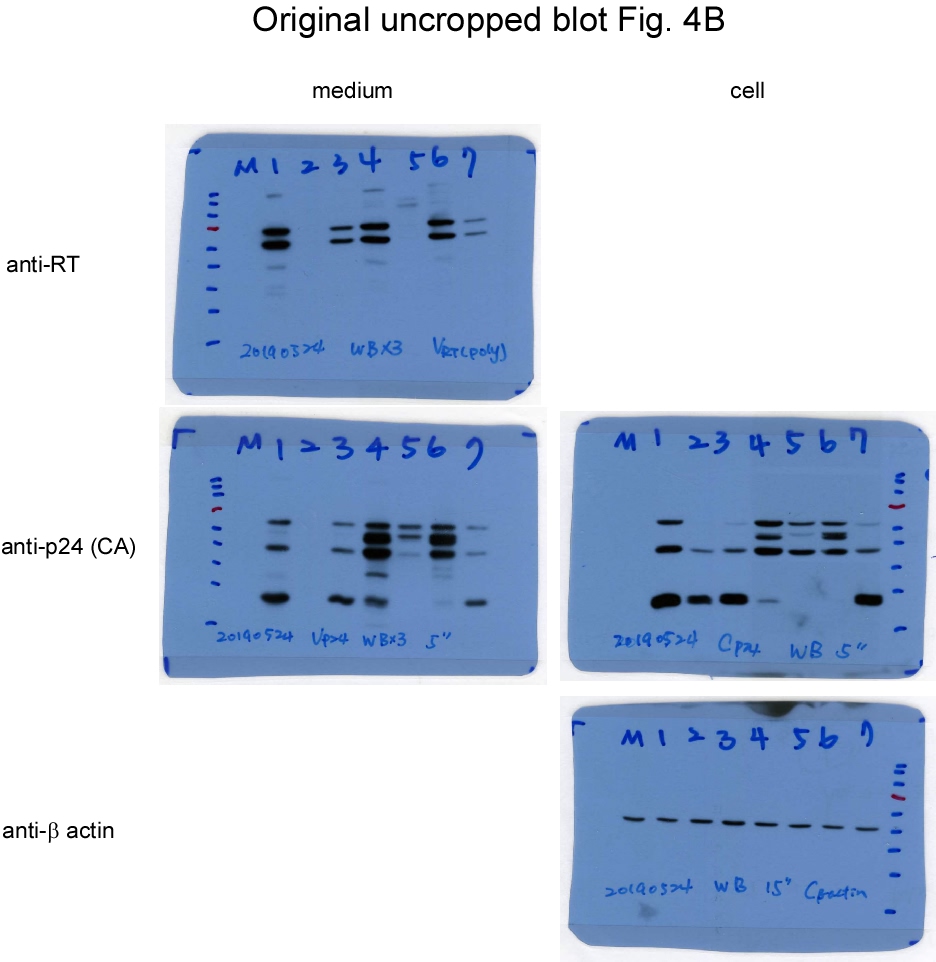

Supplement: Supplementary file 4 — Additional file 4. Original uncropped blot images for Fig. 4. [file 12866_2022_2503_MOESM4_ESM.jpg]

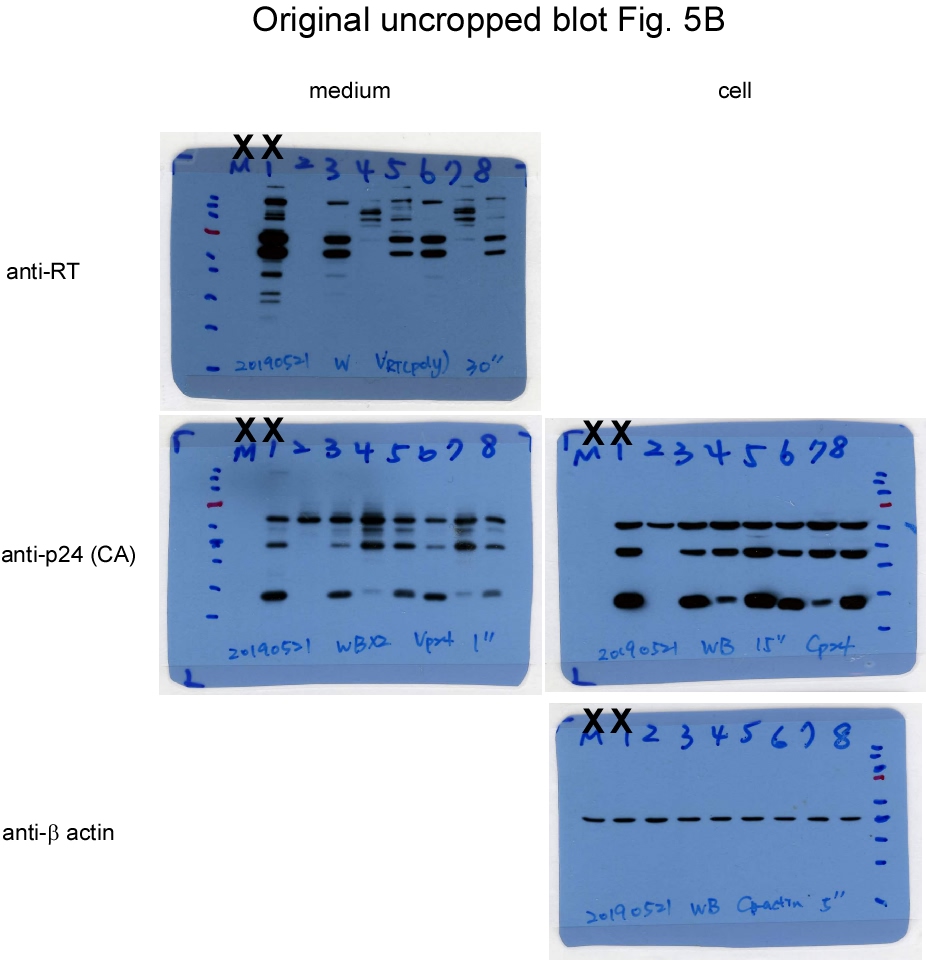

Supplement: Supplementary file 5 — Additional file 5. Original uncropped blot images for Figs. 5b-c. [file 12866_2022_2503_MOESM5_ESM.zip › Original uncropped blot Fig 5B.jpg]

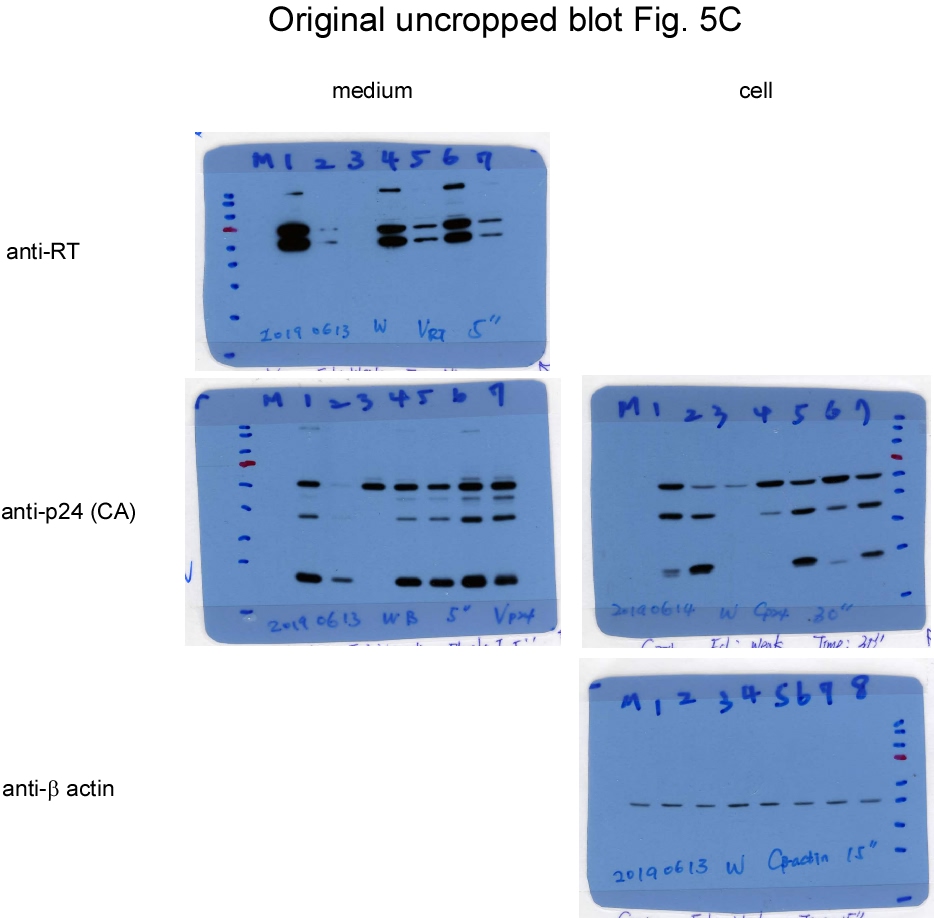

Supplement: Supplementary file 5 — Additional file 5. Original uncropped blot images for Figs. 5b-c. [file 12866_2022_2503_MOESM5_ESM.zip › Original uncropped blot Fig 5C.jpg]
